# “Much Madness is Divinest Sense”

**DOI:** 10.3201/eid1407.000000

**Published:** 2008-07

**Authors:** Polyxeni Potter

**Affiliations:** * Centers for Disease Control and Prevention, Atlanta, Georgia, USA

**Keywords:** Anne Adams, Maurice Ravel, art science connection, primary progressive aphasia, emerging infectious diseases, art and music, *Pi*, π, neurologic disorders, art and medicine, about the cover

**Figure:**
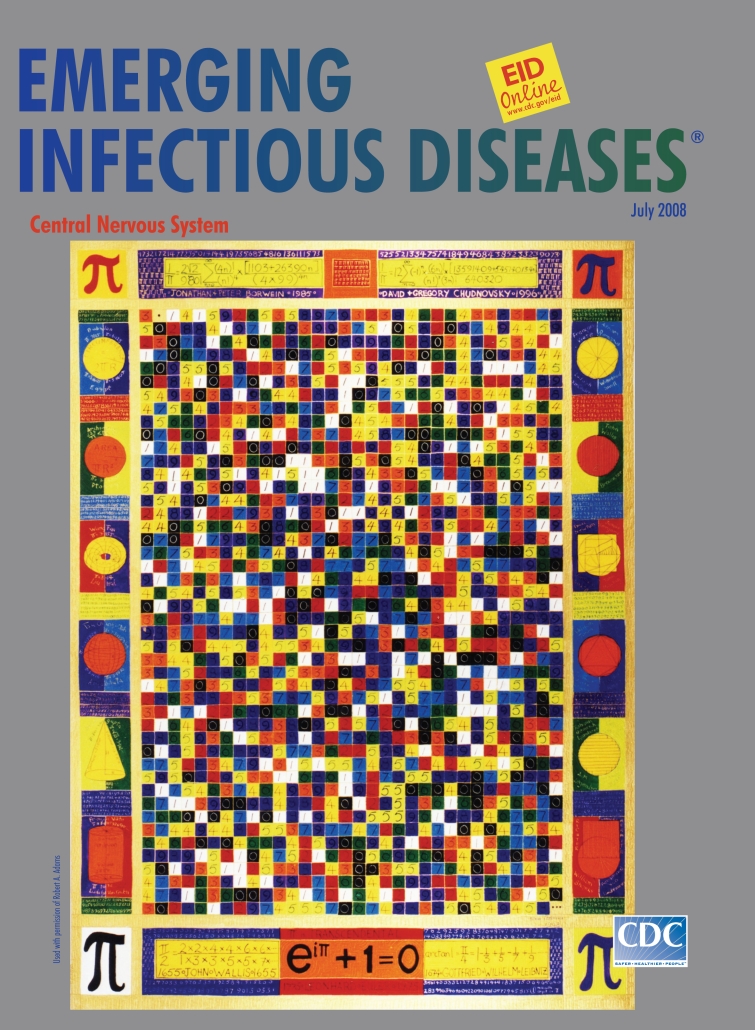
**Anne Adams (1940–2007). *Pi* (1998).** Gouache on paper. Used with permission of Robert A. Adams.

––Emily Dickinson

“And yet I still had so much music in my head,” lamented Maurice Ravel (1875–1937) near the end of his life (*1*). The French composer was frustrated by symptoms of an undiagnosed neurologic disorder that interfered with his ability to move, speak, or express creative ideas. Now labeled primary progressive aphasia-related illness, the disorder also marked the life and art of Anne Adams.

A native of Canada, Adams was educated in the sciences and excelled in physics and chemistry, which she taught at the college level. During an interval from academe, she raised her four children then returned to the sciences at age 35 as a student of cell biology, professor, and researcher. At age 46, she left again, this time to nurse her son who had been injured in an automobile wreck. The injury resolved much faster than anyone expected, but Adams decided not to return to science but pursue other interests. A lover of music and the arts, she had dabbled with painting in her earlier years, mostly architectural drawing and watercolor in a classical style.

Over the next few years, she became increasingly absorbed with art, devoting all her days to work in her studio. Her style and technique evolved rapidly, and she started experimenting, particularly with expression of sounds as visual forms. She interpreted musical scores and converted them to colorful images (Rondo alla Turquoise, Rhapsody in Blue). She became fascinated with the music of Maurice Ravel, particularly his one-movement orchestral piece Boléro.

“Don’t you think this theme has an insistent quality?” Ravel asked his friend Gustave Samazeuilh as he fingered the initial melody on the piano, “I’m going to try and repeat it a number of times without any development, gradually increasing the orchestra as best I can” (*2*). This he did. Two melodic themes were repeated eight times over 340 bars. Volume and instrumentation increased along with two alternating staccato bass lines. There was no key change until the 326th bar, when the piece accelerated into a collapsing finale (*3*). The result was haunting and infectious, an exercise in compulsion, some said perseveration.

Ravel wrote this his best-known composition while on vacation in the south of France. He was 53. Though the musical scores were marred with spelling errors, he was not yet incapacitated by illness. The success of Boléro, which he had assessed as “a piece for orchestra without music,” surprised him. During the premier of the work, a woman was said to exclaim that the composer was mad. Ravel later remarked that she must have understood the piece (*4*).

Anne Adams knew nothing of Ravel’s illness or her own. But at age 53, she started to work on the painting Unraveling Boléro, a visual analysis of Ravel’s composition. She transformed the music into colorful figures, one for each bar. Highly structured and rendered with meticulous detail, they resembled spiky space-age lace hung out to dry in neat monotonous rows. The height of figures corresponded with volume, the shape with note quality, the color with pitch.

In *Pi*, on this month’s cover of Emerging Infectious Diseases, painted when Adams was 58 and before any symptoms of language loss, she moved away from translating music toward abstraction. At the peak of her creativity, she painted mathematical concepts. And it is not surprising that she chose to paint π, one of the most mysterious and recognizable numbers, even to those who have long forgotten what it represents or how frequently it turns up in science and nature. Inside an iconic border summarizing the history of π, Adams portrayed a 32- × 46-digit portion in a matrix of the first 1,471 digits (plus the decimal point). With white, black, and component colors of the white light spectrum marking each integer from 0 to 9, she tried to capture the randomness of π’s expansion.

Loss of language (difficulty with grammar, syntax, articulation, speech) and motor function (declining muscle control), main symptoms of Adams’ (and Ravel’s) illness, have long been known to neurologists as the result of lesions on the left frontal lobe. What was extraordinary in these two cases was the simultaneous increase in capabilities of the posterior right region of the brain. Ravel died at 62 of complications after neurosurgical treatment, Adams at 67 of aspiration pneumonia brought on by severe motor and respiratory decline.

Neuropathy, with its dreaded sequelae, is a common prospect for an aging population, and not only as it relates to primary progressive aphasia. Meningitis, the scourge of children and youth as well as the immunocompromised, has multiple infectious causes and disastrous outcomes when left undiagnosed and untreated. The epidemiology of bacterial meningitis around the world keeps evolving, impeding vaccine development (*5*). Illness caused by emerging pathogens (e.g., *Rickettsia felis*) is likely underreported (*6*). Meanwhile interspecies hybrids of pathogenic yeasts that can cause meningoencephalitis (e.g., *Cryptococcus neoformans* and *C*. *gattii*) are now found in patients with weakened immune systems (*7*).

Unlike Adams and Ravel, most patients with neurologic disorders experience no increased creative powers. They face a degenerative clinical course and early death. But the spark of genius, even when ignited by illness, may shed light on unexplored areas of the mind, although how the brain supports the creative process remains as much a mystery as π.

Mathematicians and artists alike have turned to repetition and exquisite detail in their search for perfection. And so have public health researchers. Exhaustive reporting and integration of surveillance data can identify specimens for genetic analysis and clarify variants associated with susceptibility to central nervous system disease (*8*).
